# Prevalence of intestinal parasites among HIV/AIDS patients attending Infectious Disease Hospital Kano, Nigeria

**DOI:** 10.11604/pamj.2014.17.295.3707

**Published:** 2014-04-18

**Authors:** Ebenezer Feyisayo Jegede, Esther Tinuade Ibijoke Oyeyi, ArmaYau Hamisu Bichi, Henry Akwen Mbah, Kwasi Torpey

**Affiliations:** 1 Family Health International (FHI 360), Nigeria; 2 Department of Zoology, Faculty of Science, Bayero University Kano, Nigeria

**Keywords:** HIV/AIDS, CD4+ count, intestinal parasites

## Abstract

**Introduction:**

Intestinal parasitic infection has been a major source of morbidity in tropical countries especially among HIV patients. The aim of this study was to determine prevalence of intestinal parasites and its association with immunological status and risk factors among HIV infected patients in Kano, Nigeria.

**Methods:**

105 HIV+ subjects and 50 HIV- controls were recruited into the studies from June to December 2010. Clinical information was collected using a questionnaire. Single stool and venous blood samples were collected from each subject. Stool examination and CD4+ count were performed.

**Results:**

Prevalence of intestinal parasites was 11.4% and 6% among the HIV+ and control subjects respectively with no statistically significant difference (p = 0.389). Specifically, the following intestinal parasites were isolated from HIV+ subjects: *Entamoebahistolytica* (5.7%), hookworm (3.8%), *Entamoeba coli* (1%), *Blastocystishominis* (1%). Only *Entamoebahistolytica* was isolated among the control subjects. The mean CD4+ count of HIV+ and control subjects was 287 cells/ul and 691 cells/µlrespectively while the median was 279(Q1-120, Q3-384) cell/µl and 691(Q1-466, Q3-852) cell/µlrespectively with statistically significant difference (P= 0.021). Diarrhea and the absence of anti-parasitic therapy seem to be important risk factors associated with the occurrence of intestinal parasites among HIV+ subjects. A higher prevalence (14.5%) of intestinal parasites was observed in subject with CD4+ count 350cell/µl.

**Conclusion:**

Routine examination for intestinal parasites should be carried out for better management of HIV/AIDS patients.

## Introduction

According to UNAID and WHO joint report, an estimated 35.3 million people were living with HIV globally at the end of 2012 with 69% of these persons living in Sub-Saharan Africa[1]. Opportunistic infection poses major health problems among HIV patients particularly in the late stage of the disease when immune- suppression is severe.

Intestinal parasitic infection has been a major source of morbidity in tropical countries especially among HIV patients[2–5]. Diarrhea due to intestinal microbial infections is a frequent manifestation among HIV infected patients. It has been speculated that HIV infected patients may have unique types of intestinal infections, and that activation from such parasites may affect the progression of HIV disease[6].

Initiating treatment and laboratory monitoring of HIV/AIDs patients involve series of laboratory investigations, such as CD4 count, hematology and biochemistry. However, in Antiretroviral Treatment (ART) programs information on the intestinal parasitic status of HIV patients is not readily available despite its clinical importance.

There are some reports on the distribution of intestinal parasites among different communities in Nigeria[2, 7, 8]. Unfortunately, there is inadequate information on the prevalence of intestinal parasitic infection among HIV/AIDS patients,although improved diagnosis techniques to indentify opportunistic intestinal parasites are readily available in Nigeria. This study sought to determine the prevalence of intestinal parasitic infection among HIV/AIDS patients at Infectious Disease Hospital, (IDH) Kano. It also examined their immune status and some predisposing factors of acquiring intestinal parasites.

## Methods

### Study population, design and sample size

Infectious Disease Hospital, Kano, a 250-bed hospital, located in SabonGari Kano in the North West region of Nigeria. IDH Kano is a comprehensive ART site since 2005. Presently, about 12,000 patients are enrolled in IDH Kano for ART. Sample collection and clinical –epidemiologic questionnaire were performed between June 2010 and December 2010.

This was a cross sectional comparative study. Random sampling technique was used to select 105 consenting HIV positive subjects. The selection of participants was based at interval of three for each consenting participants. The sample size required for this study was calculated using the formula N = z2Pq/d2 as previously described [9]based on a prevalence of 7.29% for intestinal parasites in Kano[10].

Where:

N = number of samples Z = Confidence interval – 95% = 1.96 Kano prevalence of similar study was 7.29% [10] P = Kano state HIV prevalence rate, q = (1-p) d= Allowable error taken to be 5% (0.05). N = (1.96)2 x 0.0729 x (1-0.0729)/ (0.05)2= 3.8416 x0.0729 (0.9271)/ (0.05)2= 103.85

Thus, a minimum sample size of 103.85 was required for the study. Only 50 controls ware recruited through consecutive sampling of consenting HIV negative subjects at the HIV Testing and counselling (HTC) unit. Each study participant responded to a questionnaire on medical history, sexual behavior and basic socio-economic characteristics.

### Specimen collection and analysis

**Stool for intestinal parasites:** Single stool sampleswere collected from each participant intosterile wide mouth screw cap labeled containers and analyzed within 24 hours of collection. They were analyzed by direct wet mount[11], formal-ether concentrated method and modified ZiehlNeelsen staining[12]to detect cysts, oocyst, ova, larvae andtrophozoites of parasites.

**Blood for CD4 count:** Five milliliters of venous blood samples were aseptically drawn into labeled EDTA tubes and mixed properly to avoid blood clots. CD4 count was done within six hours of sample collection by flow cytometry method using PARTEC Cyflow[13].

**Data Analysis:** Data generated was entered into Microsoft excel 2007 cleaned and imported into Statistical Package for Social Science (SPSS) software version 16 for analysis. Differences in proportions of HIV positive patients with CD4 values within specific ranges and stool examination results showing characteristic findings was tested using chi-square test. A

**Ethical Approval:** Ethical approval was given by Kano State Hospitals Management Board in Nigeriaand Family Health International's Office of International Research Ethics in North Carolina, USA.

## Results

### Basic characteristics of studied population

A total of 105 HIV positive subjects (57 female and 48 male) with a mean age of 34.4 years and 50 HIV negative subjects (15 females and 35 males) with a mean age 31.2 years as control were evaluated in this study (Table 1). More than 80% in both groups were Hausa by tribe. The majority (61.9%) of the HIV positive subjects had no formal education compared to only 20% in the control group. Similarly, a greater proportion (57%) of the HIV positive subjects were unemployed compared to (14%) in the control group.

**Table 1:** Baseline characteristics of studied population

Features	HIV positive	HIV negative	Total
**Sex**	N (%)	N (%)	
Male	48 (48.7)	35 (70)	83
Female	57 (54.3)	15 (30)	72
**Total**	105	50	155
**Age mean(range)**	34.4(20-65)	32.1(18-67)	
18-27	29 ( 27.6 )	17 ( 34 )	
28-37	41 ( 39.1 )	22 ( 44 )	
38-47	23 ( 21.9 )	7 ( 14 )	
48-57	10 ( 9.5)	2 ( 4 )	
58-67	2 ( 1.9 )	2 ( 4)	
**Educational Status**			
Educated	40 (38.1)	40 (80)	
Un Educated	65 (61.9)	10 (20)	
**Marital status**			
Divorced	19 (18.1)	1 (2)	
Married	52 (49.5)	26 (52)	
Single	10 (9.5)	22 (44)	
Widowed	24 (22.9)	1 (2)	
**Ethnic group**			
Hausa	86 (81.9)	45 (90)	
Igbo	9 (8.6)	2 (4)	
Kogi	3 (2.9)	1 (2)	
TIV	6 (5.7)	0 (0)	
Yoruba	1 (1)	2 (4)	
**Occupation**			
Business	2 (1.9)	3 (6)	
Civil servant	11 (10.5)	6 (12)	
Lab technicians	1 (1)	1 (2)	
Farmer	5 (4.8)	3 (6)	
House wife	12 (11.4)	0 (0)	
Trader	8 (7.6)	9 (18)	
Mechanic	3 (2.9)	2 (4)	
Unemployed	60 (57.1)	7 (14)	
Student	1 (1)	13 (26)	
Tailor	2 (1.9)	1 (2)	

### Prevalence of intestinal parasite

The overall prevalence of intestinal parasites was 11.4% in HIV positive subjects, which did not present significant difference when compared to (6%) HIV negative controls (Table 2). Four different species of intestinal parasites arranged in order of prevalence, *Entamoebahistolytica (5.7%),Ancylostomaduodenale (3.8%), Entamoeba coli* (1%) and *Blastocystishominis* (1%) were recovered from the HIV positive subjects. Only one species, *Entamoebahistolytica* (6%) was found among the control group.

**Table 2:** Prevalence of intestinal parasites and risk factors analysis among HIV patients

Characteristic	No tested	No with infection (%)	OR	95%CI	P-Value
**HIV status**					
**HIV Patients**	105	12(11.4)	2.02	0.55,7.43	0.389
**Non HIV subjects**	50	3(6)	0.49	0.12,1.64	
**Sex (HIV patients)**					
**Male**	48	4(8.3)	0.56	0.16,1.95	0.54
**Female**	57	8(14)	1.8	0.51,6.30	
**Non-HIV**					
**Male**	35	2(5.7)	0.85	0.08,9.47	1
**Female**	15	1(6.7)	1.18	0.11,13.16	
**CD4+ count (cells/µl) of HIV patients**					
<350	76	11(14.5)	4.74	0.6,37.19	0.172
≥350	29	1(3.5)	0.21	0.03,1.66	
**Clinical manifestation of HIV patients**					
Diarrhea	20	9(45)	22.36	5.3,93.58	0.00
Without Diarrhea	85	3(3.5)	0.04	0.01.0.19	
**ART status**					
ART	61	5(8.2)	0.47	0.14,1.58	0.234
NON-ART	44	7(15.9)	2.12	0.63-7.10	
**Anti-parasitic Drug**					
No	7	3(42.9)	7.42	1.58,34.76	0.031
Yes	98	9(9.2)	0.13	0.03-0.63	
**Toilet Types**					
Open field	38	5(13.2)			
In House	12	0(0)	NA		
Public Toilets	55	7(12.7)			0.474
					
**Source of Drinking Water**					
Tap Water	53	7(13.2)			
Water Tank	9	0(0)	NA		
Well	43	5(11.6)			0.951
**Contact with pet/animals**					
No	75	9(12)	1.23	0.31,4.79	
Yes	30	3(10)	0.81	0.21-3.18	0.818

Legend: No- number, OR- Odd ratio, 95% CI- 95% Confidence interval, P-Value –significant when P < 0.05

In both groups, the prevalence was higher in females than males. Among HIV positive subjects it was 14% in females and 8% in males. For the control group it was 6.7% in females and 5.7% in males. Concerning immune status of subjects the mean CD4 + count among HIV positive subjects was 287cell/µl (range 12-1067) cell/µl, while control group presented 691cell /µl ( range 134-1409) cell/µl with a statistical significant difference between the two groups (P = 0.021). The median and interquartile range of CD4+ count among both groups was 279cell/µl (Q1- 120,Q3- 384) for HIV infected while 691cell/µl (Q1-466, Q3-852) was recorded among HIV negative control group. A higher percentage (72.3%) of the HIV positive subjects had a CD4+ cell count below the 350 cell/µl threshold recommended by WHO for ART initiation in developing countries. With only one exception, all cases of parasitic infection were found among HIV positive subjects with CD4+ count lower than 350cell/µl,although regression analysis did not show a significant association (Table 2).

The risk factors associated with transmission of intestinal parasitic infection in HIV infected subjects is presented in Table 2. The prevalence was lower (8.2%) among HIV infected subjects on ART compared to those not on treatment (16%). The prevalence ofintestinal parasites was lower (9.2%) among HIV infected subjects on anti-parasitic therapy compared to those not on anti-parasitic therapy (43%), which was significantly associated to acquiringintestinalparasitic infection showed by regression analysis (Table 2) The prevalence of intestinal parasitic infection was higher (45%) in HIV infected subjects presenting with diarrhea compared to those without this symptoms (3.5%). Regression analysis also showed diarrhea to be a predictor of intestinal parasites amongHIV patients. The prevalence was about 13% when the toilet type used was either open field or public toilet and zero among in-house toilet users.

Based on the source of drinking water, the prevalence among consumers of tap water was (13.2%), well water was (11.6%), but was absent among tank water consumers. The assessment of contact with pets/animals revealed similar prevalence rates (10% and 12%) among the contact and non- contact groups respectively.

An incidental finding, following stool examination, was the presence of numerous pus cells among 7.6% of the HIV positive patients with CD4+ count as low as 29 cell/µl. Pus cells were absent in the stool of HIV negative control individuals.

## Discussion

In this study, we investigated the prevalence of intestinal parasitic infection among HIV seropositive individuals and its association withimmune status in IDH Kano Nigeria. This study detected non-opportunistic intestinal parasites in 11.4% of HIV positive study participants. The prevalence was lower (6.0%) among HIV negative control subjects. Four different speciesnamely, *Entamoebahistolytica* (5.7%), *Ancylostomaduodenale* (3.8%) *Blastocystishominis* (1%) *Entamoeba coli* (1%) were isolated from the HIV positive subjects. It is noteworthy that among the 20 HIV positive subjects presenting with diarrhea, the prevalence of non-opportunistic intestinal parasites was 45%. Thus, clinical diarrhea seems to be strongly associated with presence of intestinal parasites among these subjects.

We observed numerous pus cells in the stool of highly immune-compromised HIV positive subjects. Further microbiological investigations would be required to identify the specific pathogens triggering this reaction and the relationship with HIV/AIDS, immune status and diarrhea.

Based on the method used for parasite concentration and detection in this study, the intestinal parasites burden may have been underestimated. Probably because, other diagnostic techniques for specific parasites concentration and identification such Kato- katz, Bearman's method, floatation technique and microsporidium spore staining were not used. The prevalence of parasitic infection (11.4%), reported among HIV infected patients in this study was lower than that found in similar studies in Nigeria reporting a prevalence above 24% [2, 8, 14].

The absence of opportunistic intestinal parasites in this study is similar to the prevalence reportedin other studies among HIV positive patients in Lagos [15] and Enugu [16] in Nigeria. Converselya prevalence rate of about 25% *for Cryptosporidium* species was reported in Jos [7] and Abeokuta [17] in Nigeria. Higher prevalence (31.4%) of opportunistic intestinal parasites were found among HIV patients suffering from diarrhea in Korea [18]. One of the possible reason for this variation could be that majority (71%) of HIV infected patients did not have contact with pets or other animals which are potential sources of transmission of oocyst of coccidians parasites. Another reason could be thatthe study participants are enrolled into the ART program, managed and monitored by trained ART physicians, and placed on Cotrimoxazole therapy according to the national guidelines [19].

In our study, intestinal protozoan pathogens were detected more frequently in cases with CD4+ count [20, 21]. In this study, we found *Entamoebahistolytica* (5.7%) to be the most prevalent parasite in HIV positive subjects. This is in conformity with previous studies done in Abuja byUdeh et al[14] where the highest prevalence obtained for this parasite was 36.79%. In contrast, *Cryptosporidium parvum* has been reportedas the most common parasite in HIV infected persons [8]. Previous studies conducted in Nepal[22] and Iran [23] reported *Crytosopridiumparvum* and *Giardia lambliaas* commonest parasites among HIV infected persons and most of the times were associated with diarrhea. In this study, it was observed that females had higher prevalence (14%) of intestinal parasitic infection than males (8.3%) among HIV positive subjects with no significant difference (P > 0.05). This is consistent with a report from Abeokuta, Nigeria [2]. Diarrhea and absence of anti-parasitic therapy are the most likely factors for predicting the acquiring of common intestinal parasites among HIV infected patients.

Diarrhea is a common symptom in HIV infectionand a major signs to AIDS progression with possibility of various opportunistic infections, however, none of the opportunistic coccidian was identified in this study. Nevertheless this fact is in conflicts with other reports in Nigeria [7, 8].

The study had some potential limitations. The control group does not match the cases, as it was difficult to recruit apparently healthy persons in this hospital setting. In addition, stool examination was done only once, which may not have given an accurate picture of intestinal parasite prevalence.

## Conclusion

In conclusion, *Entamoebahistolytica, Ancylostomaduodenale* and *Blastocystishominis* are the most common intestinal pathogens among HIV infected patients in IDH Kano. Diarrhea and absence of anti-parasitic therapy are the most likely factors for predicting the acquiring of common intestinal parasites among HIV infected patients, These findings add conceptual importance and relevance to the association between intestinal parasites and HIV infection. Thus routine stool examination should be considered as an important component in ART monitoring program in Nigeria.
